# Automated Intra-abdominal Pressure Monitoring During Orthotopic Heart Transplant Leads to Early Diagnosis and Treatment of Intraoperative Abdominal Compartment Syndrome—A Case Report

**DOI:** 10.3389/fsurg.2022.812288

**Published:** 2022-02-28

**Authors:** Manxu Zhao, Nicola D'Attellis, Dominic Emerson, Vanessa Moll, Fardad Esmailian

**Affiliations:** ^1^Department of Anesthesiology, Cedars-Sinai Medical Center, Los Angeles, CA, United States; ^2^Department of Cardiac Surgery, Smidt Heart Institute, Cedars-Sinai Medical Center, Los Angeles, CA, United States; ^3^Potrero Medical Inc., Hayward, CA, United States; ^4^Department of Anesthesiology, Emory University School of Medicine, Atlanta, GA, United States

**Keywords:** intra-abdominal pressure, abdominal compartment syndrome, heart transplant (HTx), retroperitoneal hematoma, continuous monitoring, extracorporeal membrane oxygenation (ECMO), intra-abdominal hypertension (IAH)

## Abstract

We describe a case of spontaneous retroperitoneal hematoma leading to abdominal compartment syndrome and organ failure during a complicated orthotopic heart transplantation in a patient previously on mechanical circulatory support. After the patient had been weaned of cardiopulmonary bypass, the patient suddenly became hemodynamically unstable despite good LV and RV function. While the patient was resuscitated, high intra-abdominal pressures were noted on a novel monitor measuring real-time intra-abdominal pressures and urinary output. The early detection of high intra-abdominal pressures led to a swift decompressive laparotomy with the detection of retroperitoneal hematoma and subsequent hemodynamic stabilization.

## Introduction

Intra-abdominal hypertension (IAH) and subsequent abdominal compartment syndrome (ACS) have been associated with increased morbidity and mortality (1, 2). Risk factors for IAH and ACS include massive transfusion or fluid resuscitation, hypothermia, and acidosis (1). In patients with high clinical suspicion of IAH, Intra-abdominal pressure (IAP) is measured intermittently, which is challenging during a surgical procedure. Intravesicular IAP measurement is considered the gold standard using an instillation volume of 25 mL sterile saline with the transducer zeroed at the mid-axillary line. IAP should be measured at end-expiration, with the patient in the supine position and ensuring that there is no abdominal muscle activity. The ability to automatically monitor bladder pressure (and derived IAP) through a Foley catheter was not possible until FDA recently approved the Accuryn Monitoring System (Potrero Medical, Hayward, CA) for IAP, urine output, and temperature monitoring.

In this report, we describe a rare case of spontaneous retroperitoneal hematoma leading to ACS with severe hemodynamic instability during an orthotopic heart transplant (OHT). The patient had been on mechanical circulatory support [extracorporeal membrane oxygenation (ECMO) and Impella CP (Abiomed)] going into the surgery. Early detection of elevated intra-abdominal pressures was facilitated using the automated real-time IAP monitoring.

## Case Report

A 58-year-old male with a 5-year history of non-ischemic cardiomyopathy [left ventricular ejection fraction (LVEF) 10%, severe right ventricular (RV) dysfunction, automated implantable cardioverter-defibrillator (AICD)] presented to an outside hospital with acute decompensated heart failure and an episode of ventricular tachycardia (VT). He was subsequently transferred to our institution for a higher level of care and evaluation for heart transplantation. His past medical history was significant for non-ischemic cardiomyopathy, multiple episodes of VT, paroxysmal atrial fibrillation, hypertension, and polycystic kidney disease with stage III chronic kidney disease. Baseline laboratory values included brain natriuretic peptide (BNP) 4,495 pg/ml (normal <100 pg/ml), creatinine 1.92 mg/dL (normal 0.72–1.25 mg/dl), and total bilirubin 5.7 mg/dL (normal 0.2–1.1 mg/dl).

A few hours after arrival at our institution, the patient experienced another episode of VT which was terminated by anti-tachycardia pacing without AICD shocking. Amiodarone and furosemide infusions were initiated for antiarrhythmic and diuretic treatment. A transthoracic echocardiogram showed LVEF 9.8% calculated by Simpson's biplane with left ventricular internal diameter end diastole (LVIDd 2D) of 7.5 cm (normal 4.2–5.9 cm) and tricuspid annulus plane systolic excursion (TAPSE) of 1.1 cm (normal 1.6–3.0 cm). A pulmonary artery catheter was placed in the right internal jugular vein revealing pulmonary artery pressure (PAP) of 84/53 mmHg (mean PAP 68 mmHg), pulmonary capillary wedge pressure of 45 mmHg, and aortic pressure of 106/83 mmHg with mean of 92 mmHg. Left and right coronary catheterization revealed normal coronary arteries. With VT prohibiting high dose inotropic support, a right femoral intra-aortic balloon pump was placed for afterload reduction with 1:1 augmentation synchronized by pressure trigger. Computed tomography (CT) chest and abdomen as part of pre-heart transplant evaluation revealed cardiomegaly with a severely dilated left heart and pulmonary edema without intrinsic lung disease. Despite hemodynamic improvement and a decreasing mPAP to 45–55 mmHg with combined diuresis and IABP support, the patient continued showing signs of hypoperfusion with lactate increasing from initial 2.8–5.1 mmol/L (normal 0.5–2.2 mmol/L), creatinine of 2.48 mg/dL and low mixed venous oxygen partial pressure of 31 mm Hg (normal 35–45 mm Hg), The patient was then escalated (same day) to femoral-femoral veno-arterial extracorporeal membrane oxygenation (VA-ECMO) with Impella placement to decompress a dilated left ventricle while the IABP was removed. MAP improved with a decrease in PAP and CVP upon VA-ECMO initiation. TEE revealed mildly improved RV function with stable volume status and normalized mixed venous oxygenation from 31 mmHg to 49 mmHg. Proper Impella position was confirmed by TEE. Combined ECMO and Impella support gradually improved the patient's hemodynamics, reflected in normalizing laboratory parameters. The patient was extubated on day 3 while on ECMO/Impella support and listed for heart transplantation. On day 6, an organ became available, and the patient underwent an OHT using a bicaval approach. At the time the patient was treansported to the OR VA-ECMO provided a flow of 4.4 L/min, pump speed of 3,040 RPM with FiO_2_ of 1.0. The Impella was set at 1.5 L/min with a purge flow of 10.9 ml/hr. Unfractionated heparin was purged through the Impella at 600 units/h with an activated clotting time (ACT) goal of 160–180 s.

**Figure 1:**
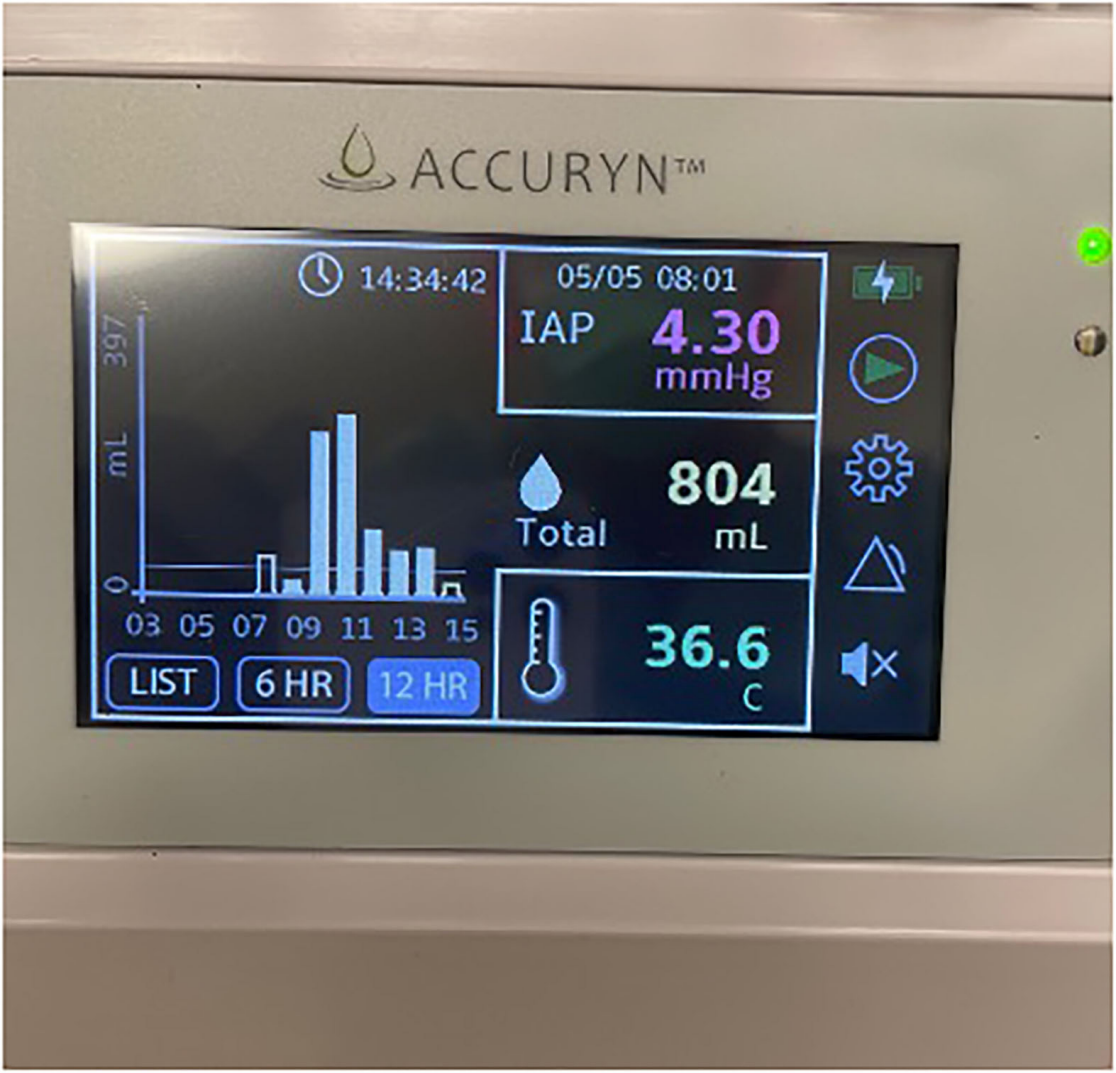
Photo of the Accuryn Monitoring System's display showing urine output, temperature, and intra-abdominal pressure. (Note that this picture was not taken during the patient's surgery. It serves as an example of this monitoring system).

**Figure 2:**
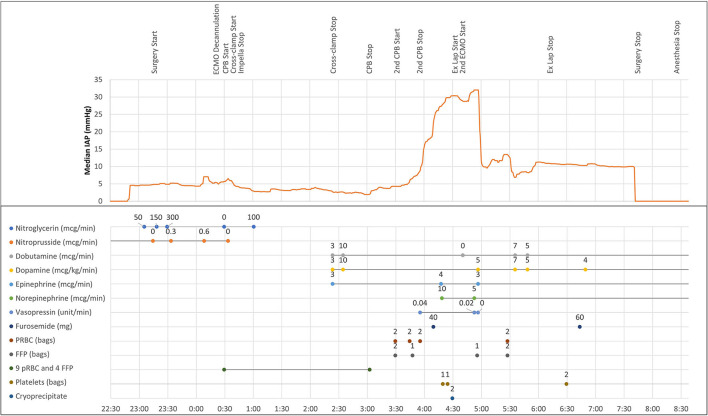
Intraoperative intra-abdominal pressure trendline and clinical timeline, including vasoactive medications and transfusion of blood products. Intra-abdominal pressure measurements were downloaded from the Accuryn Monitoring System. ECMO, extracorporeal membrane oxygenation; CPB, cardiopulmonary bypass; ex lap, exploratory laparotomy; pRBC, packed red blood cells; FFP, fresh frozen plasma.

Following uneventful induction of general anesthesia, a bladder catheter measuring automated urine output and frequent intra-abdominal pressure (IAP) (Accuryn Monitoring System, Potrero Medical, Hayward, CA, Figure 1) was inserted, as per the institution's standard. VA-ECMO was converted to a cardiopulmonary bypass (CPB) circuit, and the left femoral artery and venous cannulas were removed. The Impella catheter was removed from the right groin with subsequent femoral artery repair, and the patient underwent an OHT in the standard fashion. During the 149 min of CPB, the patient required significant blood product transfusions with 9 units of pRBC and 4 units of FFP. The patient was weaned off CPB without difficulty with BP of 110/72 mmHg, PAP of 31/13 mmHg, and CVP of 5 mmHg. Transesophageal echocardiogram (TEE) showed the transplanted heart with mildly reduced LVEF and a mild to moderately depressed RV. However, the patient's hemodynamic status rapidly deteriorated with the lowest BP reaching 59/43 mmHg despite active fluid resuscitation with 4 units of pRBC and 4 units of FFP while maintaining CVP at 7–10 mmHg and mean PAP at 20–25 mmHg. Inotropic and vasopressor support were escalated to dopamine at 10 mcg/kg/min, dobutamine at 10 mcg/kg/min, epinephrine at 4 mcg/min, and norepinephrine at 10 mcg/min, vasopressin at 0.04 unit/min. During ongoing resuscitation, an IAP of 32 mmHg was measured by the Accuryn Monitoring System (see IAP trend, Figure 2). The cardiac surgeon was notified, and the clinical examination revealed a significantly tensed abdomen. The patient was then quickly placed back on central VA-ECMO via the ascending aorta and the right atrium (48 min after weaning from CPB) for hemodynamic support while addressing ACS management. Vascular surgery was consulted, and an emergent exploratory laparotomy was performed. During laparotomy, a large retroperitoneal hematoma and diffuse bleeding were identified with no obvious source. The patient's IAP decreased to 10 mmHg (see Figure 2) immediately upon performing the laparotomy, and the patient's hemodynamic status stabilized despite ongoing bleeding that was addressed with the transfusion of 4 units pRBC. Inotropic and vasopressor support was titrated down (dopamine at 5 mcg/kg/min, dobutamine at 4 mcg/kg/min, epinephrine at 3 mcg/min, norepinephrine 4 mcg/kg, and vasopressin off). The patient was then transferred to the intensive care unit on central ECMO with an open abdomen and chest. On post-operative day (POD) 1, continuous renal replacement therapy was initiated (creatinine peaked at 2.4 mg/dL). On POD 2, VA-ECMO was successfully weaned and removed, and the chest and abdomen closed. The patient improved gradually and was extubated on POD 7. Renal function also gradually improved, and dialysis was discontinued on POD 40. The patient's medical condition continued to improve with normal biventricular function and mild to moderate tricuspid regurgitation assessed by TTE. The patient was discharged home on POD 65.

## Discussion

This report highlights the benefit of frequent automated IAP monitoring in complex cardiac surgery patients with associated risk factors for IAH or ACS. Intraoperative ACS was detected early due to the high-fidelity IAP monitoring system and successfully treated with decompressive laparotomy. To our knowledge, this is the first case of early intraoperative ACS detection using real-time IAP monitoring.

Intra-abdominal hypertension is defined by a sustained or repeated pathological elevation of IAP ≥12 mmHg (1). Abdominal compartment syndrome is the final stage of a continuum of physiologic derangements resulting from the sustained elevation of IAP exceeding 20 mmHg leading to newly diagnosed organ dysfunction and failure. ACS can be divided into primary, secondary, and recurrent. Primary ACS refers to an intra-abdominal cause, such as in this case, retroperitoneal hematoma. Secondary ACS has an extra-abdominal cause such as massive fluid resuscitation leading to intraperitoneal edema. The World Society of Abdominal Compartment Syndrome (WSACS) consensus guidelines state that IAP should be measured at end-expiration in the complete supine position after ensuring that abdominal muscle contractions are absent. Grading of IAH follows a continuum of sustained increased IAP per WSACS consensus guidelines as shown in Table 1 (1). The management algorithm for IAH/ACS addresses five therapeutic interventions: evacuation of intraluminal contents, evacuation of intra-abdominal space-occupying lesions, improvements of the abdominal wall compliance, optimization of fluid administration and the optimization of systemic and regional perfusion. Decompressive laparotomy should be considered when ACS is refractory to medical management. While the overall quality of evidence available guiding the development of WSACS recommendations was low (1), abdominal decompression can be lifesaving when ACS is refractory to medical treatment or as in our case developed rapidly and should then be performed expeditiously.

**Table 1:** World Society of Abdominal Compartment Syndrome (WSACS) consensus definitions on the grading of IAH and ACS.

**IAH Grades**	**IAP (mmHg)**
IAH grade I	12–15
IAH grade II	16–20
IAH grade III	21–25
IAH grade IV	>25
ACS	>20 with new onset of organ dysfunction or failure

*IAP, intra-abdominal pressure; IAH, intra-abdominal hypertension; ACS, abdominal compartment syndrome*.

Cardiac surgical patients have several risk factors for developing IAH or ACS, such as (intraoperative) hypothermia, massive fluid resuscitation, and multiple transfusions (1, 3). Anticoagulation necessary for CPB or ECMO poses another risk factor for spontaneous intra-thoracic, intra-abdominal or retroperitoneal hemorrhage. In the context of anticoagulation, the predominant hypotheses of spontaneous retroperitoneal hematoma include diffuse small vessel arteriosclerosis, heparin-induced immune microangiopathy, and unrecognized minor trauma (4–6). While intraoperative IAH during cardiac surgery has rarely been reported, the incidence of post-operative IAH is reported to be as high as 46% (7–9). However, none of these patients progressed to ACS, possibly due to underpowering of these studies (138 and 69 patients, respectively) (7, 9). The incidence and outcomes of perioperative ACS in cardiac surgical patients are less well-described (10–13). Recently, a prospective observational multicenter study reported one percent of cardiac surgery patients developed ACS post-operatively and require decompressive laparotomy, with significant associated mortality of 57% (13). We could only identify two prior case reports describing the development of intraoperative ACS during cardiac surgery: (1) secondary ACS during failure to maintain CPB while undergoing elective minimally invasive right mini-thoracotomy mitral valve and tricuspid valve repairs (14). In this report, the authors reported a “relatively long period of time was needed to make a diagnosis of ACS” as automated other potential causes of hemodynamic instability were assessed (14). (2) Rabbi et al. described another secondary ACS occurring during elective coronary revascularization, resulting in the inability to wean off CPB (10). In both cases, clinical suspicion for ACS was eventually raised due to a tense and distended abdomen. In the latter case report, bladder pressure was measured manually, demonstrating an IAP of 50 mmHg (10). Both patients were successfully weaned off CPB after decompressive laparotomies were performed.

Few small studies describing the incidence of IAH during and after cardiac surgery have been published (11, 15–18). Although in these studies, no patient progressed to ACS, it is important to note study size may have been too small with 138 and 69 patients, respectively (7, 9).

Clinical manifestations of ACS are direct consequences of IAH, leading to a decrease in abdominal perfusion pressure (mean arterial pressure minus IAP) which leads to organ dysfunction (19). IAH may cause visceral organ hypoperfusion leading to acute kidney injury, intestinal ischemia, bacterial translocation, the release of cytokines, and production of free oxygen radicals, contributing to the development of multiple organ failure in critically ill patients (11, 19–21). In addition, IAH reduces cardiac output, increases systemic vascular resistance, pulmonary artery pressure, and pulmonary artery wedge pressure (17). In our case, as a result of retroperitoneal hemorrhage, our patient became rapidly hemodynamically unstable demonstrating severe hypotension, despite higher inotrope and vasopressor support. Automated IAP measurement led to the early diagnosis of ACS, resolved by an exploratory laparotomy with immediate relief of IAP from 32 to 10 mmHg (Figure 2).

WSACS recommends serial trans-bladder measurement of IAP (at least every 4 h) to detect IAH and avoid progression to ACS in patients with one or more risk factors or with clinical suspicion of IAH (1). While intermittent manual monitoring of IAP during surgery is usually not feasible, automated monitoring displaying IAP values and trends is now available using this novel device. During the case, the Accuryn Monitoring System calculated IAP every 30 seconds from bladder pressure and provided during the 508 minutes of surgery a detailed course of IAP.

This case report describes the use of automated IAP monitoring in a high-risk and complex patient undergoing OHT, for early intraoperative detection and treatment of ACS. Ultimately, automated IAP monitoring, early detection of ACS, and subsequent treatment of ACS improved clinical outcomes for this patient.

## Summary

IAH and ACS have been associated with increased morbidity and mortality. Early detection of IAH and ACS is necessary for optimal patient outcomes. Cardiac surgery patients display many risk factors for IAH/ACS including massive transfusion or fluid resuscitation, hypothermia, and acidosis. In this case report, IAP monitoring in a high-risk and complex patient who underwent an orthotopic heart transplant procedure led to intraoperative recognition and treatment of ACS.

## Data Availability Statement

The original contributions presented in the study are included in the article/Supplementary Material, further inquiries can be directed to the corresponding author/s.

## Ethics Statement

Written informed consent was obtained from the individual for the publication of any potentially identifiable images or data included in this article.

## Author Contributions

MZ wrote the initial draft, reviewed, and edited the manuscript. ND'A, DE, and FE reviewed and edited the manuscript. VM reviewed, revised, and edited the manuscript. All authors agree on this submission.

## Conflict of Interest

VM is currently employed by Potrero Medical. The remaining authors declare that the research was conducted in the absence of any commercial or financial relationships that could be construed as a potential conflict of interest.

## Publisher's Note

All claims expressed in this article are solely those of the authors and do not necessarily represent those of their affiliated organizations, or those of the publisher, the editors and the reviewers. Any product that may be evaluated in this article, or claim that may be made by its manufacturer, is not guaranteed or endorsed by the publisher.
